# Clinical and ultrasound characteristics in patients with sars-cov-2 pneumonia, associated with hospitalization prognosis. e-covid project

**DOI:** 10.1186/s12890-024-03439-2

**Published:** 2024-12-31

**Authors:** Noemí Fàbrega Ramon, Marta Ortega Bravo, Gerard Torres Cortada, Joaquim Sol Culleré, Mònica Solanes Cabús, Jose María Palacín Peruga

**Affiliations:** 1 Centre d’Atenció Primària Onze de Setembre. Gerència Territorial de Lleida, Institut Català de La Salut, Passeig 11 de Setembre,10 , 25005 Lleida, Spain; 2 Fundació Institut Universitari Per a La Recerca a L’Atenció Primària de Salut Jordi Gol i Gurina (IDIAPJGol), Barcelona, Spain; 3 University of Lleida, Lleida, Spain; 4 Grup de Recerca d’ecografia Clínica en Atenció Primària (GRECOCAP Group), Fundació Institut Universitari Per a La Recerca a L’Atenció Primària de Salut Jordi Gol i Gurina (IDIAPJGol), Gran Via de Les Corts Catalanes, 587, 08007 Barcelona, Spain; 5 Centre d’Atenció Primària d’Almacelles, Melcior de Guàrdia, Gerència Territorial de Lleida, Institut Català de La Salut, Barcelona S/N 25510 Almacelles, Spain; 6 Hospital Universitari Santa María. Gerència Territorial de Lleida, Institut Català de La Salut, Barcelona, Spain; 7 Translational Research in Respiratory Medicine. Biomedical Research Institute of Lleida (IRBLleida), Lleida, Spain; 8 CIBER of Respiratory Diseases (CIBERES), Institute of Health Carlos III, Madrid, Spain; 9 Family Phisician, Executive Board of the Catalan Society of Family and Community Medicine (CAMFiC), 08009 Barcelona, Spain

**Keywords:** Ultrasound, SARS-CoV-2, Hospitalization, Prognosis, Primary health care

## Abstract

**Background:**

During the COVID-19 pandemia, the imaging test of choice to diagnose COVID-19 pneumonia as chest computed tomography (CT). However, access was limited in the hospital setting and patients treated in Primary Care (PC) could only access the chest x-ray as an imaging test. Several scientific articles that demonstrated the sensitivity of lung ultrasound, being superior to chest x-ray [Cleverley J et al., BMJ 370, 202013] and comparable to CT scan [Tung-Chen Y et al., Ultrasound Med Biol 46:2918-2926, 2020], promoted the incorporation of this technique in the assessment of COVID-19 patients in PC. [Pérez J et al., Arch. Bronconeumol 56:27-30, 2020; Gargani L et al., Eur Heart J Cardiovasc Imaging 21:941-8, 2020, Soldati G et al., J Ultrasound Med 39:1459, 2020] A prior study in our territory (Lleida, Spain) was designed to predict complications (hospital admission) of COVID-19 pneumonia in PC patients, being different patterns of Lung ultrasounds (LUS) risk factors for hospital admission. [Martínez Redondo J et al., Int J Environ Res Public Health 18:3481, 2021] The rationale for conducting this study lies in the urgent need to understand the determinants of severity and prognosis in COVID-19 patients with interstitial pneumonia, according to its lung ultrasound patterns. This research is crucial to provide a deeper understanding of how these pre-existing ultrasound patterns related to disease progression influence the medical treatment.

**Methods:**

The objective of the study is to generate predictive models of lung ultrasound patterns for the prediction of lung areas characteristics associated with hospitalizations and admissions to the Intensive Care Unit (ICU) associated with COVID-19 disease, using ultrasound, sociodemographic and medical data obtained through the computerized medical history.

**Results:**

A single relevant variable has been found for the prediction of hospitalization (number of total regions with potentially pathological presence of B lines) and one for the prediction of ICU admission (number of regions of the right lung with potentially pathological presence of B lines). In both cases it has been determined that the optimal point for classification was 2 or more lung affected areas. Those areas under the curve have been obtained with good predictive capacity and consistency in both cohorts.

**Conclusions:**

The results of this study will contribute to the determination of the ultrasound prognostic value based on the number of lung areas affected, the presence of pulmonary condensation or the irregularity of pleural effusion patterns in COVID-19 patients, being able to be extended to other lung viral infections with similar patterns.

**Supplementary Information:**

The online version contains supplementary material available at 10.1186/s12890-024-03439-2.

## Background

At the end of December 2019, the first cases of a new coronavirus disease (COVID-19) were detected in the city of Wuhan (Hubei province, China). On January 30, 2020, the World Health Organization (WHO) declared the outbreak of SARS-CoV-2 in China, the causative agent of COVID-19, a public health emergency at international level [1]. Its rapid arrival in Spain forced to adapt the activity in the Primary Care (PC) from March 2020 to face up the wave of patients with COVID-19, [2] which was known to cause a disease with a wide clinical spectrum, ranging from mild symptoms to severe involvement with respiratory failure [3–11]. During the pandemic, globally, the imaging test of choice to diagnose COVID-19 was chest computed tomography (CT). However, access was limited to the hospital setting and patients treated in Primary Care could only access the chest x-ray as an imaging test. The publication of several scientific articles that demonstrated the sensitivity of Lung Ultrasounds (LUS), being superior to chest x-ray [12, 13]and comparing it in a study to CT scan [14], promoted the incorporation of this technique in the assessment of COVID-19 patients in some health care areas [15–17], mainly in Primary Care Health System, where the introduction of ultrasound was considered an important point to promote. Furthermore, LUS seem to report additional advantages, complications and the risk of hospitalization could be predicted based on the patterns observed in the lung ultrasound performed by a general practitioner. In addition, the identification of demographic, clinical, and hospital-level risk factors that may be associated with death, in critically ill patients with COVID-19 can facilitate the identification of candidates to specific medications and supportive therapies aimed to improve outcomes [18]. A prior study carried out in our territory (Lleida, Spain), in which LUS was evaluated to predict complications in primary care patients with COVID-19 pneumonia, such as hospital admission, identified different patterns of LUS (diffuse, diffuse attenuated and predominantly unilateral) as a risk factors for hospital admission [13] highlighting the predictive value of lung ultrasound in PC. Currently, the COVID-19 warning situation has ended and hospital and ICU admissions have radically decreased. The arrival of vaccination (fourth and fifth waves, 15–3–21 to 13–10–21) at the beginning of 2021 gave way to an epidemiological situation that allowed the severity and the number of cases to decrease but with significant upswings. All with important variations depending on the geographical area. Nevertheless the COVID-19 infection in PC is still present [19–21]. It is therefore considered that, although COVID-19 continues to be relevant to public health, the transition to a new sustainable COVID-19 surveillance and control strategy integrated into the surveillance and prevention of Acute Respiratory Infections (ARIs), focused on the axes presented below [22].

Globally, the number of new cases increased by 52% during the 28-day period of 20 November to 17 December 2023 as compared to the previous 28-day period, with over 850.000 new cases reported. During the period from 13 November to 10 December 2023, over 118 000 new COVID-19 hospitalizations and over 1600 new Intensive Care Unit (ICU) admissions have been recorded with an overall increase of 23% and 51% respectively amongst the countries reporting consistently within the current and past reporting periods [23, 24].

The rationale for conducting this study lies in the urgent need to understand the determinants of severity and prognosis in patients with COVID-19 interstitial pneumonia, according to its lung ultrasound patterns. This research is crucial to provide a deeper understanding of how these pre-existing ultrasound patterns, which are related to disease progression, influence the medical decision and treatment carried out, particularly in the context of covid, being able to be extrapolated to other lung viral infections.

## Methods

### Objective

The aim of the study was to generate predictive models based on lung ultrasound patterns obtained through performing a LUS in COVID-19 patients in primary care, for the prediction of hospitalizations and admissions to the hospital intensive care unit.

### Study design

A prospective cohort study design was carried out in primary care centres of Catalunya (Spain) using data obtained through lung ultrasound, sociodemographic data and previous pathologies collected.

### Study participants

Participants included during the period 05/12/2020 to 05/11/2021, had a suspected clinical diagnosis or confirmed diagnosis by reverse transcription Polymerase Chain Reaction (RT-PCR) test for COVID-19. Antigenic tests were not included since these were not yet available. Clinical lung ultrasounds were performed during the 7-day period after diagnosis, and patients who had been hospitalized for COVID-19 in the 90 days prior to the new diagnosis were excluded.

### Data collection

Sociodemographic data and previous pathologies collected in the computerized medical Electronic Health Record (EHR) from the data base system Information System for Research in Primary Care (SIDIAP) [25, 26] which captures clinical information of approximately 5,8 million Catalan citizens (around 80% of the Catalan population). This information is pseudonymised and it is originated from different data sources: Electronic History Record (EHR) in PHC of the Catalan Health Institute); including sociodemographic characteristics, comorbidities registered as International Classification of Diseases (ICD)−10 codes [27].

We collected patients’ data: sociodemographic variables (age and sex), clinical variables (symptoms, day of onset, heart rate, respiratory rate, oxygen saturation), RT-PCR test from the nasopharyngeal swab, and ultrasound analysis patterns of seriousness categorized according to the hospital guidelines. Lung ultrasound findings: + indicates < 3 lines B (normal). + + indicates > 3 lines B (mild interstitial). + + + indicates white lung (diffuse interstitial). Table 1.

**Table 1:** Lung ultrasound findings

+ / + + / + + +	B lines
yes / no	pleural irregularity
yes / no	subpleural condensation
yes / no	condensation

+ indicates < 3 lines B (normal)

+ + indicates > 3 B lines (mild interstitial)

+ + + indicates white lung (diffuse interstitial)

**Fig. 1:**
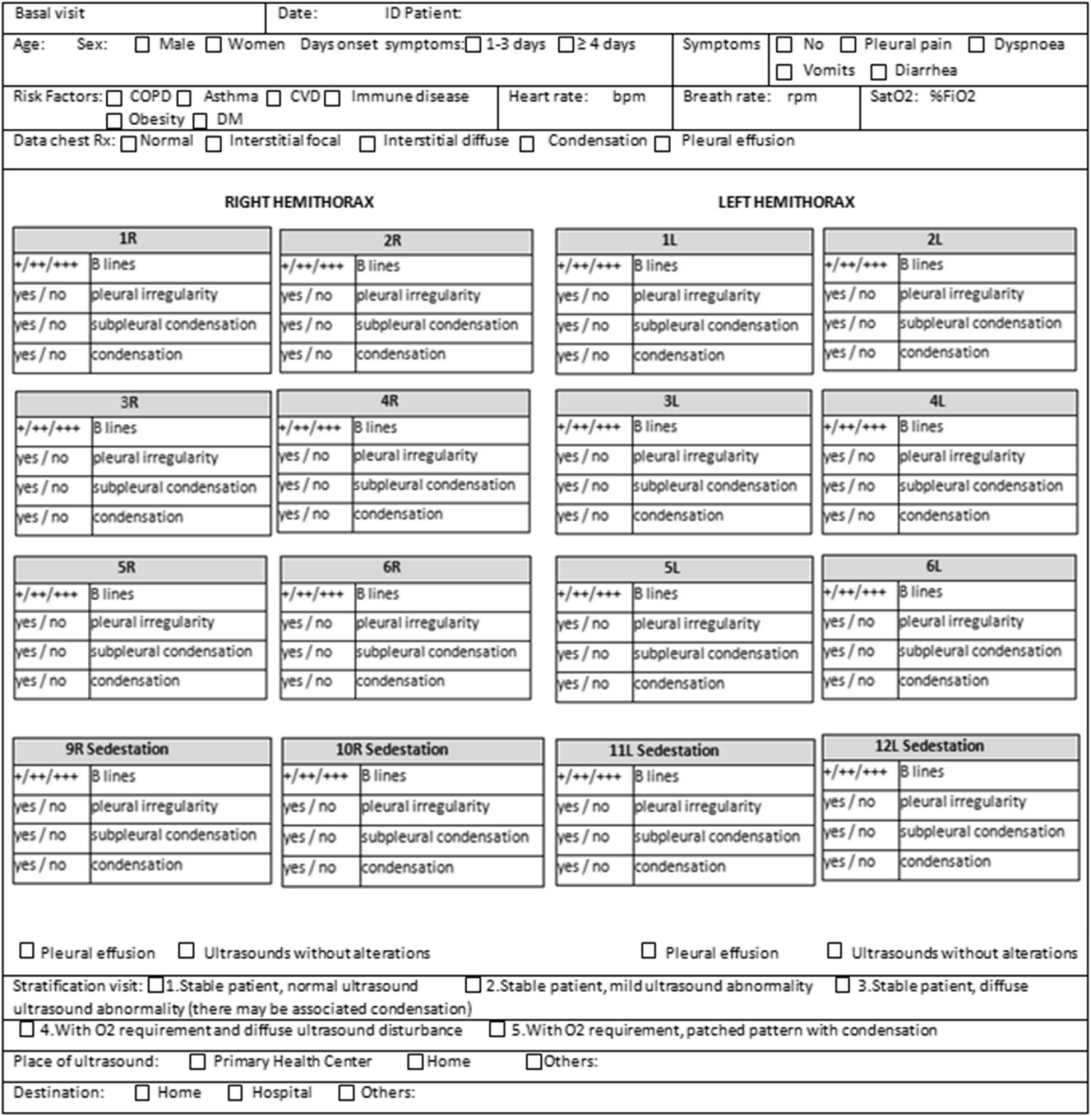
Basal variables

**Fig. 2:**
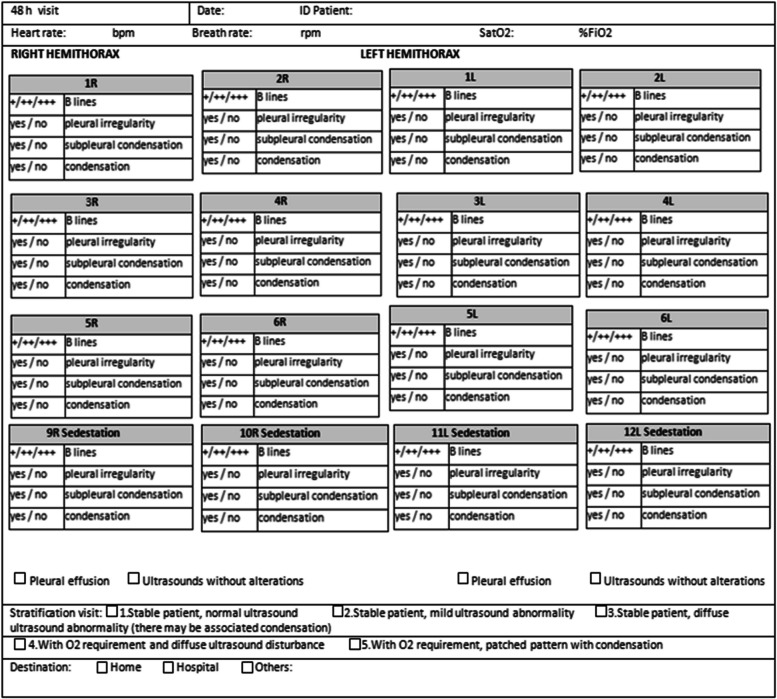
Variables at 48 h from basal visit

**Fig. 3:**
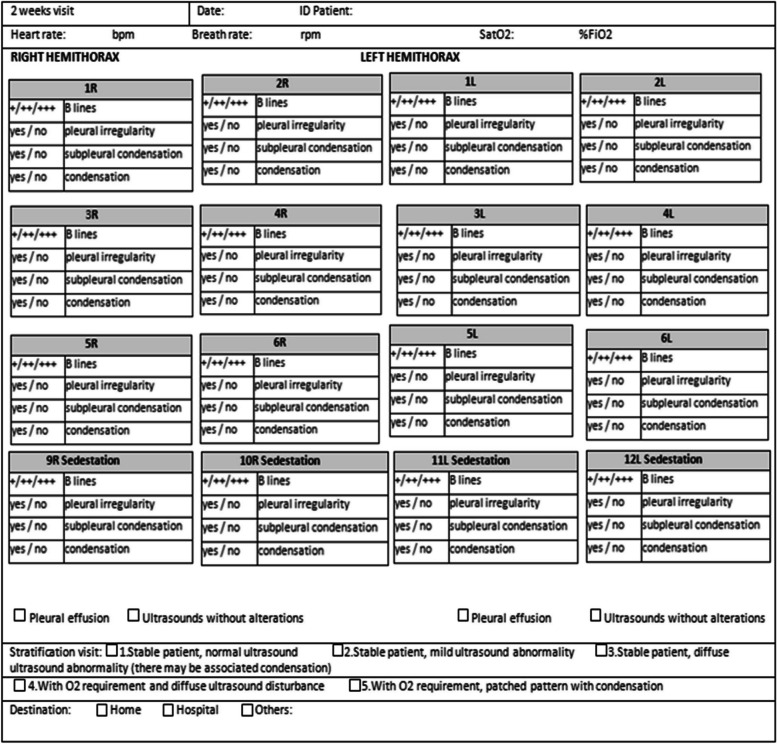
Variables at 2 weeks from basal visit

**Fig. 4:**
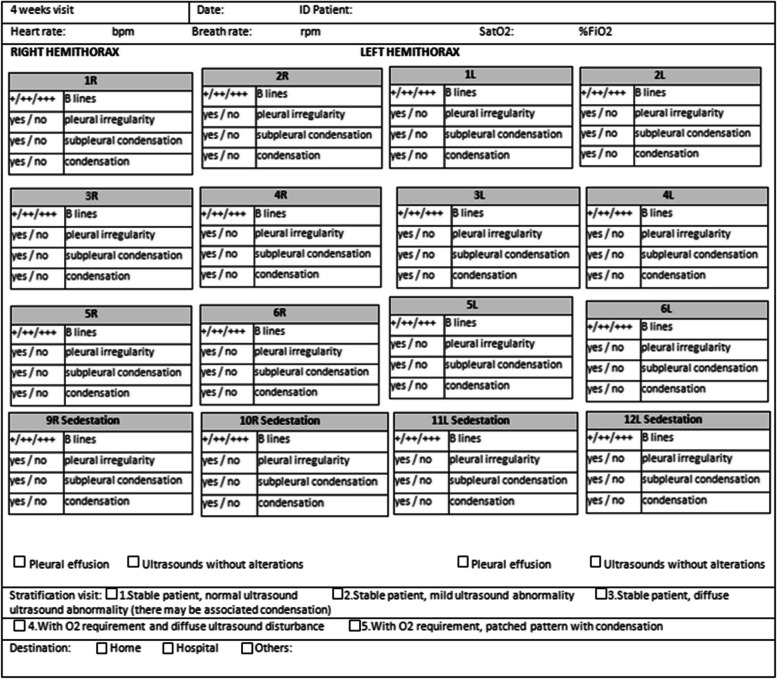
Variables at 4 weeks from basal visit

**Fig. 5:**
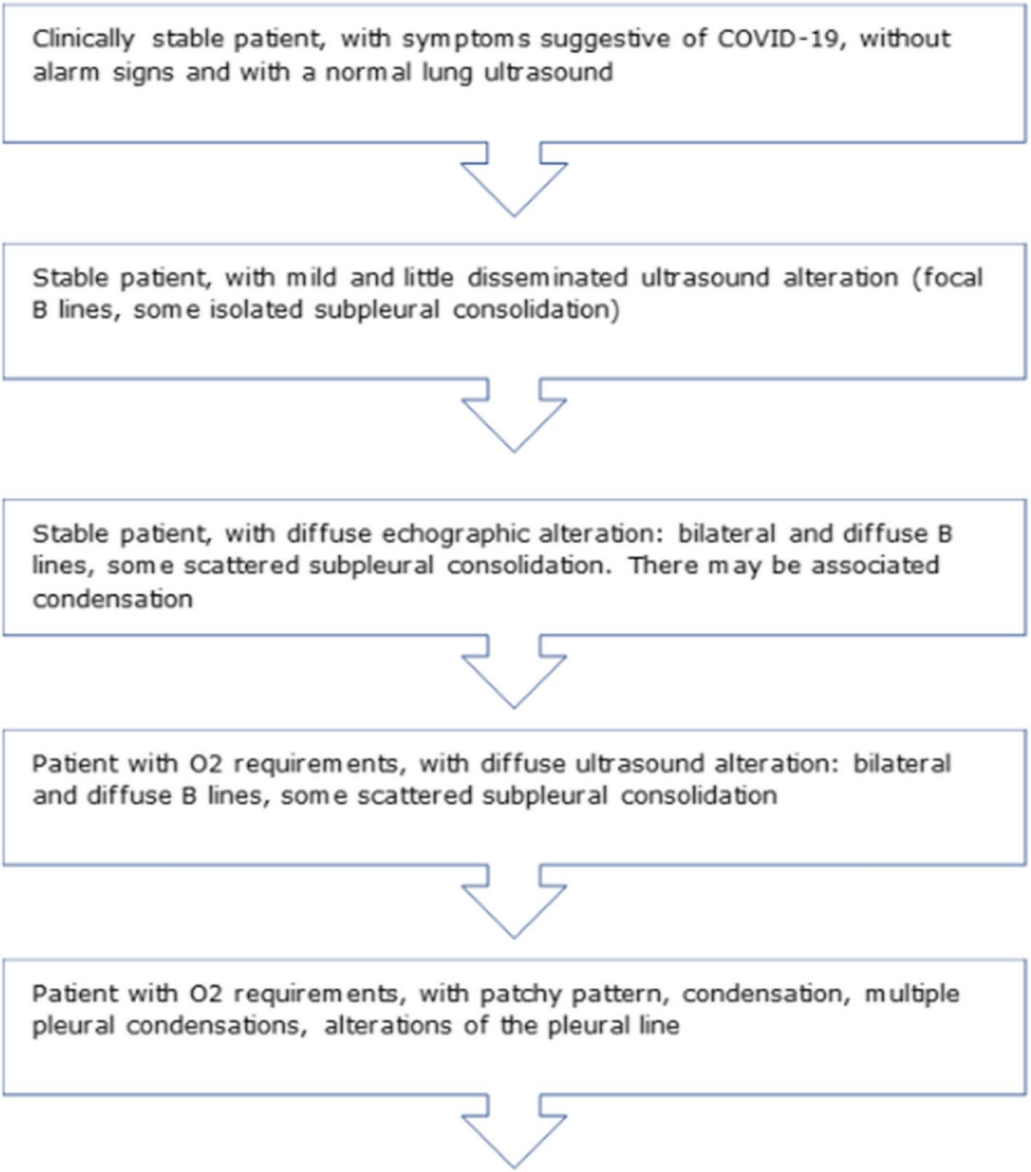
Severity findings and clinical-sonography risk stratification characterization classification provided by the Catalan Society of Family and Community Medicine (CAMFIC)

All information related to the type of injury, location and extent was recorded in the Data Collection Report (DCR). Figures 1, 2, 3 and 4. It was typified based on the ultrasound patterns and the severity. Depending on the ultrasound and clinical records at the baseline visit, 48 h, 2 weeks and 4 weeks. The clinical-ultrasound stratification was proceeded by risk. Figure 5. This characteristic was based on the classification provided by the Catalan Society of Family and Community Medicine (CAMFIC) [28].

**Fig. 6:**
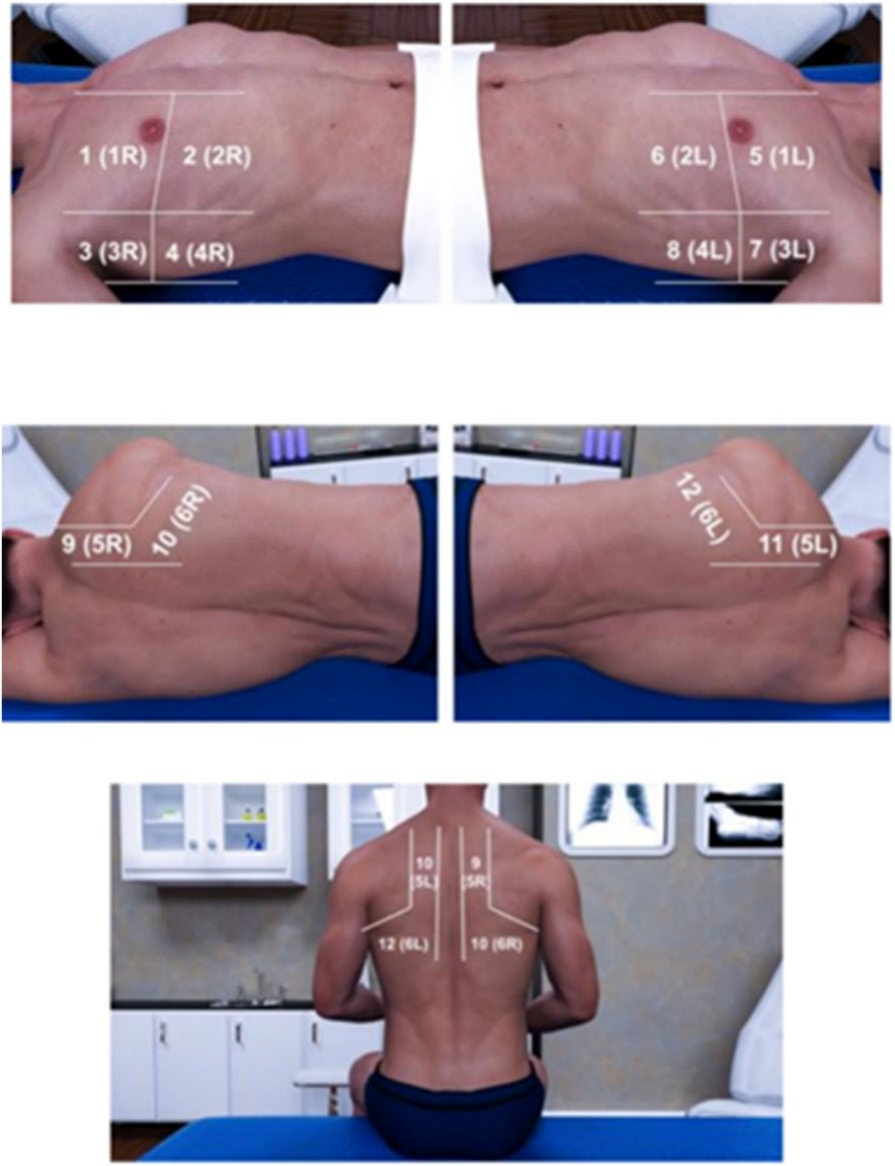
Thoracic areas studied

The lung ultrasound was conducted by scanning 4 zones (craniocaudal lines) per lung. A total of 8 zones were scanned in the posterior position (per lung: parallel and 5 cm from thoracic spine, medial border of scapula, posterior axillary line and medial axillary line). A convex probe CH5-2 (linear probe VF10-5 for children or very small-framed patients) with a bandwidth of 2–5 MHz (5–10 MHz for linear probe) was used on a Siemens Acuson X150 (Seoul, Korea) system. The exam was performed using the B mode with a general setting (pulmonary) focused at the pleural line level (1–3 cm under, in case of obese patients) and a depth set to 6–12 cm. Fig. 6.

For the generation and evaluation of the predictive models, two cohorts were considered at independent time points. A model generation and evaluation training cohort (TC) was recruited from 05/12/2020 to 05/11/2021 and a validation cohort (VC) from 05/12/2021 to 30/06/2022. A hospital admission was considered as a stay of more than 1 day in the hospital within 30 days after the ultrasound and associated with the diagnosis of COVID-19, and an ICU admission was considered as an ICU discharge record related to the hospital admission. Sex-gender analysis was incorporated into the data analysis.

### Ethical approval

The protocol was evaluated by the Clinical Research Ethics Committee (CEIC) of the Primary Care Research Institute (IDIAP Jordi Gol). Register CEIC 20/080-PCV. A copy was explained and provided full information on the project, written consent has been requested for participation in the study. Those patients who have started the circuit already existing for the diagnosis and follow-up of COVID-19 patients using ultrasound in PC, prior to the start of the study, had been contacted by telephone and written consent was requested. Informed consent was obtained from all subjects involved in the study. The variables collected were processed anonymously and guaranteed the confidentiality of data, according to what is established by Regulation (EU) 2016/679 of the European Parliament and the Council of April 27 for the protection of data (RGPD) and the organic law. 3/2018, of December 5, on the protection of personal data and guarantee of digital rights. The data base was left to the hands of the IP and the research team in an Excel format and with access using a password. For analysis, an anonymized data base was used.

### Statistical analysis

R statistical software, version 4.0.2, was used to analyse the data. To generate the models, the variables were focused and standardized, and those with near zero variance were eliminated. Dummy encoding was used for categorical variables. The variables initially considered can be seen in Supplementary Methods. A variable selection approach based on Random Forest algorithms was used using the VSURF package, focused, first, on the detection of all relevant variables for classification and, second, on the elimination of redundant variables with the purpose of optimizing data collection.

Once the models were defined, the area under the ROC curve, the optimal cut-off points (point of the ROC closest to the upper left corner), the accuracy, sensitivity, specificity, positive predictive value and negative predictive value with their respective intervals were calculated. 95% confidence interval for the training and validation cohort, using the cut-off points established in the model generation cohort. Subsequently, the temporal component was taken into account using survival models. Kaplan–Meier curves were generated and Cox regressions were generated using the cut-off points and previously calculated probabilities. Hazard Ratios and their confidence intervals from the Cox model were calculated [29].

## Results

A total of 1681 participants have been included, 1232 for Training Model (TM) and 449 for Validation Model (VM). 55.9% patients in the TM cohort were women with a mean age of 51.1 and 53.7% in the VM cohort were also women with a mean age of 49.8. In the TM, 2.19% patients received at least one dose of vaccine, unlike the 62.6% vaccinated patients in the VM statistically significant. 17.0% patients were hospitalized in the TM, 12% hospitalized in the VM statistically significant Table 2.

**Table 2:** Sample description

	Training (*N* = 1232)	Validation (*N* = 449)	*p-value*
Diagnostic ultrasound-days mean (SD)	2.40 (2.53)	2.34 (2.53)	0.708
Sex (female) n (%)	689 (55.9%)	241 (53.7%)	0.444
Age, mean (SD)	51.1 (17.6)	49.8 (18.7)	0.177
Vaccinated against COVID-19 (at least one dose)n (%)	27 (2.19%)	281 (62.6%)	< 0.001
Hospitalization n (%)	209 (17.0%)	54 (12%)	**0.017**
Admission to ICU (intensive care unit) n (%)	43 (3.49%)	10 (2.23%)	0.249
Ultrasound-hospitalization days, mean (SD)	2.19 (3.49)	2.57 (3.69)	0.495
Ultrasound-ICU days, mean (SD)	3.66 (2.17)	6.86 (4.95)	0.140
Hospitalization days, mean (SD)	11.4 (12.3)	13.0 (17.2)	0.513
Days of admission to ICU, mean (SD)	3.14 (10.9)	4.50 (13.0)	0.48

### Predictive models

**Fig. 7:**
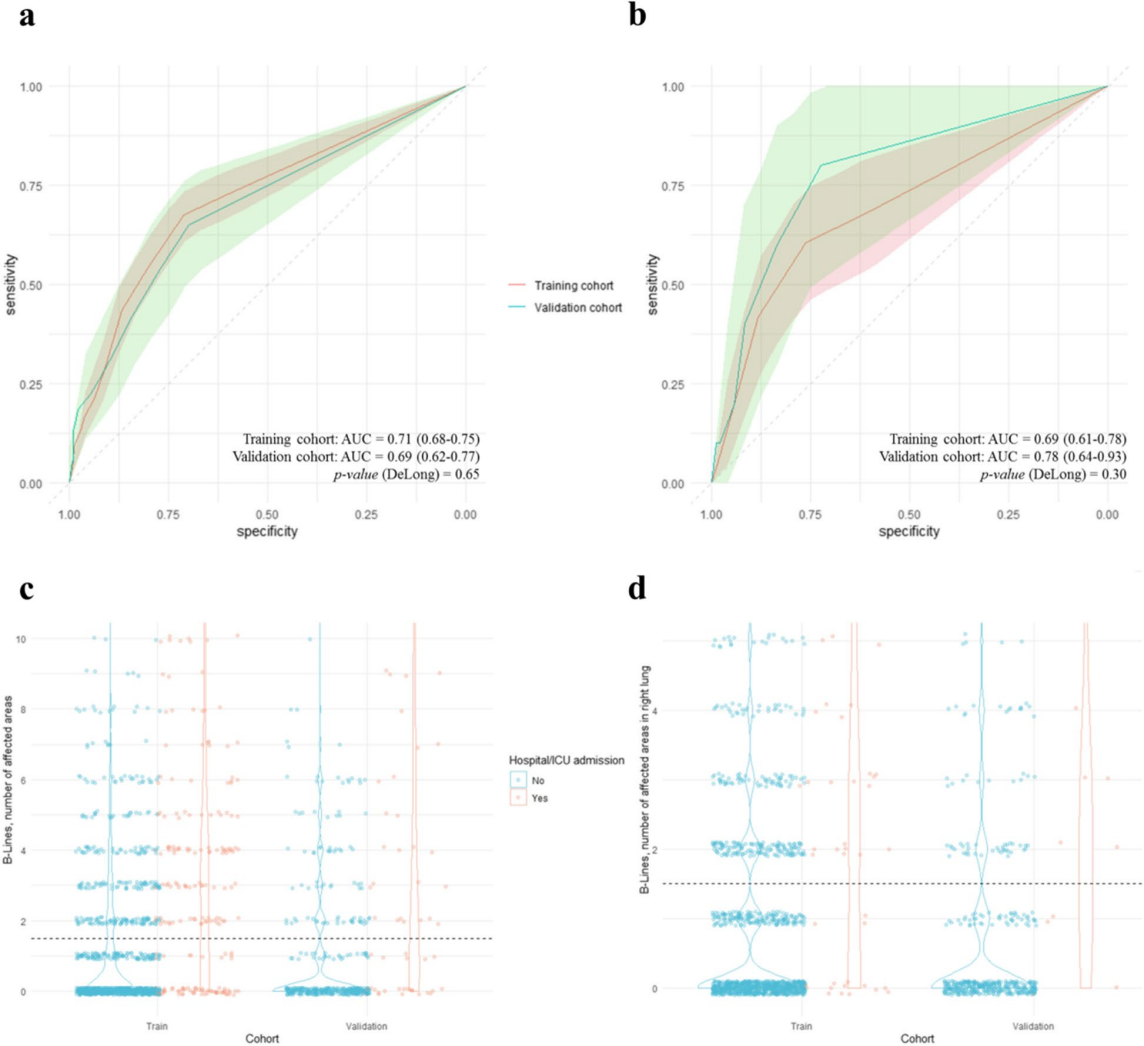
Predictive capacity for hospitalization and ICU admission based on lung ultrasound. **a** ROC curve of the hospitalization prediction model, using the total number of regions with B lines as a predictor. **b** ROC curve of the ICU admission prediction model, using the total number of regions with B lines in the right lung as a predictor. **c** Violin plot of the hospitalization prediction model, using the total number of regions with B lines as a predictor. **d** Violin plot of the ICU admission prediction model, using the total number of regions with B lines in the right lung as a predictor. The dashed horizontal lines represent the optimal cut-off point for predicting hospitalization/ICU admission

A single relevant variable has been found for the prediction of hospitalization (number of total regions with potentially pathological presence of B lines) and one for the prediction of ICU admission (number of regions of the right lung with potentially pathological presence of B lines). In both models, TM and VM, it has been determined that the optimal point for classification was 2 or more affected areas. Areas under the curve have been obtained with good predictive capacity (values around 0.7 in all cases) and consistent between both cohorts, with sensitivity and specificity values between 60 and 80% in the majority of the cases. Table 3, Fig. 7.

**Table 3:** Performance of predictive models for hospitalization and ICU admission based on lung ultrasound

	ROC AUC (95%CI)	HR (95%CI)	Cutoff	Accuracy (95%CI)	Sensitivity (95%CI)	Specificity (95%CI)	PPV (95%CI)	NPV (95%CI)
Hospital admission model performance								
Training	0.71 (0.67–0.75)	1.24 (1.20–1.29)	≥ 2	70.54 (67.9–73.07)	67.46 (61.11–73.82)	71.16 (68.39–73.94)	32.34 (27.95–36.73)	91.46 (89.52–93.4)
Validation	0.69 (0.62–0.77)	1.25 (1.17–1.35)	≥ 2	74.39 (70.09–78.36)	53.7 (40.4–67)	77.22 (73.08–81.35)	24.37 (16.66–32.08)	92.42 (89.57–95.28)
ICU admission model performance								
Training	0.69 (0.61–0.78)	1.52 (1.32–1.74)	≥ 2	75.65 (73.15–78.02)	60.47 (45.85–75.08)	76.2 (73.78–78.62)	8.41 (5.32–11.51)	98.16 (97.29–99.03)
Validation	0.78 (0.64–0.93)	1.70 (1.28–2.25)	≥ 2	82.85 (79.04–86.22)	60 (29.64–90.36)	83.37 (79.89–86.85)	7.59 (1.75–13.44)	98.92 (97.87–99.97)

For the hospitalization model, the number of ultrasound regions with potentially pathological presence of B lines has been used as a predictor variable, and for the ICU admission model, the presence only in the right lung has been used. To calculate the ROC curves and Hazard Ratios, the variable has been used as continuous, and for the rest of the calculations the optimal cut-off point of ≥ 2 has been established. ROC: Receiver Operating Characteristic, AUC: Area Under the Curve, CI: Confidence Interval, HR: Hazard Ratio, PPV: Positive Predictive Value, NPV: Negative Predictive Value, ICU: Intensive Care Unit.

### Survival models

**Fig. 8:**
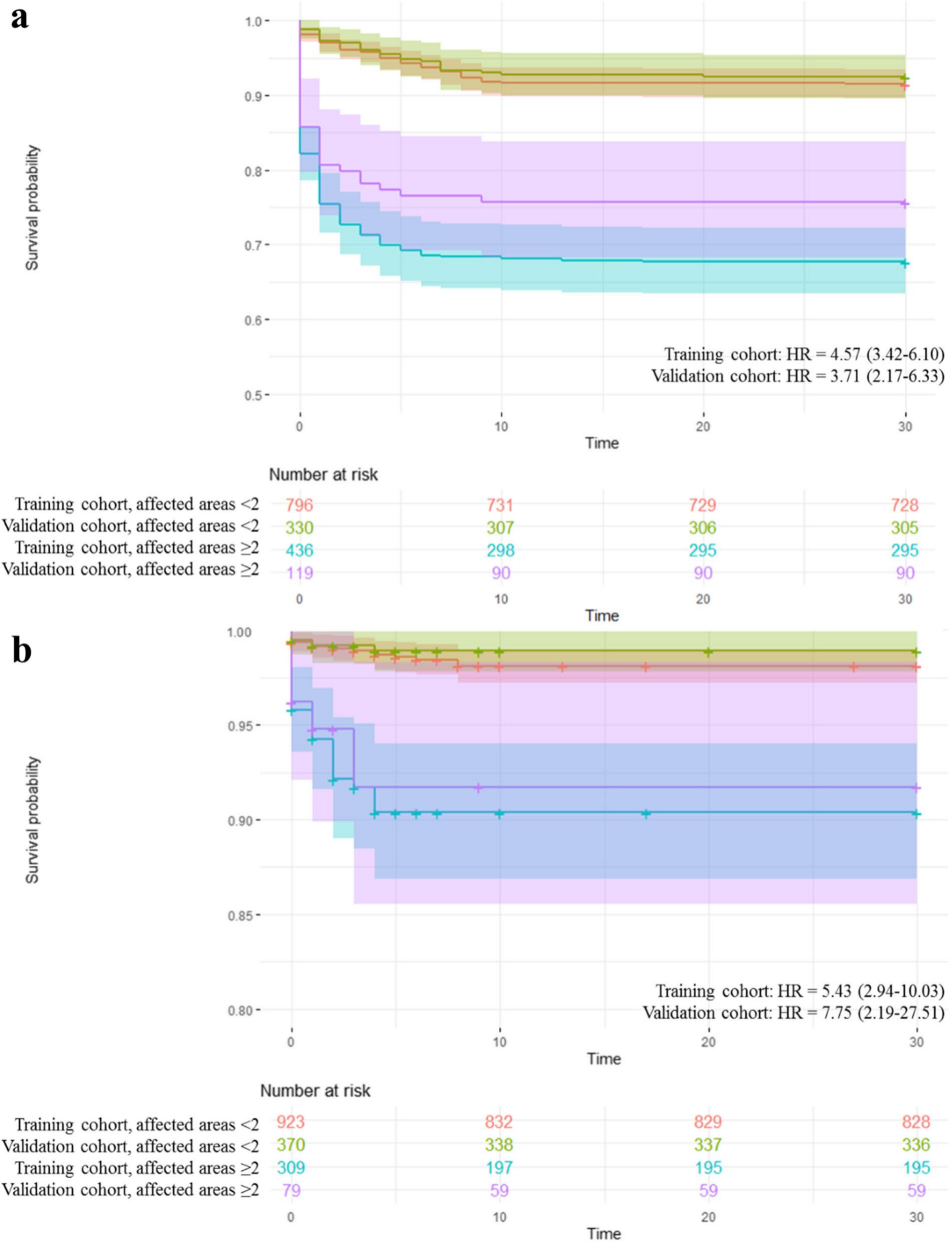
Survival models for hospitalization and ICU admission based on lung ultrasound. **a** hospitalization prediction model, using the total number of regions with B lines ≥ 2 as a predictor. **b** prediction model for ICU admissions, using the total number of regions with B lines in the right lung ≥ 2 as a predictor. HR: Hazard Ratio

Using the same predictor variables, survival models and Cox regressions have been generated. For the Cox regressions, the Hazard Ratios (HR) have been calculated, treating the variables both continuously and establishing the cut-off point of ≥ 2. Considering the variables as continuous, for each more affected area an increase in the HR for hospitalization of 1.24 in the training cohort and 1.25 in the validation cohort is observed. For the ICU admission model, HRs of 1.52 have been found in the training cohort and 1.70 in the validation cohort (Table 1). Setting the cut-off point of ≥ 2, the HR of hospitalization is 4.57 and 3.71 in the training and validation cohorts, respectively, and the HR of admission to the ICU is 5.43 and 7.75. Fig. 8.

## Discussion

The main contribution in our study, is the determination of the number of lung affected areas which have risk of hospitalization. Settle that both cohorts of patients (the validation and the training cohort), were comparable and in agreement with others reports, [30–36] it has been determined that the optimal classification point was 2 or more affected areas with pathological presence of B lines, associated with the risk of hospitalization, and for the prediction of ICU admission (number of regions of the right lung with potentially pathological presence of B lines). Other literature examined findings were at least 3 B-lines appeared independently of the number of lung areas affected [37].

According to that the presence of B lines in the lung ultrasound and the severity of the lung disease. The B lines are associated with the presence of liquid in the alveolar spaces, which is a sign of inflammation and pulmonary edema. Therefore, the detection of B lines can indicate a serious degree of pulmonary compromise and, therefore, a greater risk of hospitalization. This mean prognostic value of B-lines: The presence of B-lines on lung ultrasound has been associated with adverse outcomes in patients with various respiratory diseases, including pneumonia and acute respiratory distress syndrome (ARDS) [37]. Therefore, the identification of B lines can help identify patients who are at higher risk of developing serious complications that require hospitalization or ICU admission. Moreover, the extension of the injuries like the number of areas affected with the presence of B lines is an important factor for predicting the risk of hospitalization and admission to the ICU. This suggests that the extent of lung lesions, assessed by lung ultrasound, may be an important indicator of disease severity and the need for intensive medical care.

In our study, we noticed a right lung preference of lung lesions in COVID-19. Like in other literature, the lesions on the right lung were significantly larger and developed faster than those on the left [38]. The anatomy of the right bronchus, which has a larger diameter than the left one, could justify this greater impact of covid. Moreover, the level of right-over-left preference of lung injury was significantly correlated with the potential need for intensive care admission [38]. Some studies can explain this analysis based on the lung anatomy and structure. The right lung has three lobes (upper, middle, and lower), while the left lung has only two (upper and lower), which could make the right lung more susceptible to the spread of infection due to its greater structural complexity and volume. Moreover the right main bronchus is wider, shorter and positioned in a more vertical position compared to the left main bronchus. This also facilitates the arrival and dissemination of viral particles. On the other hand the distribution of blood flow on the right lung receives a greater volume of blood flow than the left, due to the greater perfusion of the right side of the heart [39]. This could facilitate the spread of the virus through the blood and contribute to a greater severity of the lesions in the right lung. Studies have shown that there are differences in ventilation patterns between the right and left lungs, with more favorable distribution of ventilation in the right lung under normal conditions. Viral particles have an easier way of infecting and spreading in the right lung, either through the airways due to the anatomical features of the right main bronchus or through the blood flow due to the greater perfusion on the right side. ARDS and sepsis can cause increased capillary permeability to cause pulmonary edema [40]. These patterns could affect the spread and severity of lesions in the right lung during an infection such as COVID-19. In addition, the right lung may have differences in immunological response compared to the left, which could influence the severity of the inflammatory response and lesion formation during a viral infection such as COVID-19 [41]. These arguments could help explain why more lesions are observed in the right lung in patients with COVID-19. However, it is important to note that more research is needed to fully understand the underlying mechanisms behind this observed preference.

In the survive model we observed that for each more affected area establishing the cut-off point of two or more than two lung areas, had an increase in the HR for hospitalization in the training cohort and in the validation cohort. Moreover we observed more survival in the survive model in the training and validation cohort with less than two lung areas affected, and less survival when the patients had two or more than two lung areas damaged.

These arguments suggest that the extent of the affected lung areas can significantly influence the risk of hospitalization and the frequency of clinical follow-up in patients with certain lung diseases, such as COVID-19. Patients with fewer lung areas affected may be considered lower risk and therefore may require less clinical follow-up to monitor their progression and response to treatment. On the other hand, those with more damaged areas may need closer monitoring to quickly detect and manage any clinical worsening.

These data highlight the usefulness of lung ultrasound in primary care for early diagnosis of COVID-19-related interstitial disease.

Early quantification of the severity of lung involvement in COVID-19 patients may be obtained by estimating the overall amount of lung areas detected as being pathological with ultrasound. In our study we describe the justification that more than 2 ultrasound lung areas are considered pathological. The diseased lung is identified by the presence of any pathological finding (separated and coalescent B-lines, light beams, consolidations) and the areas of diseased lung whose have a significant statistically meaning are measured 2 or more than 2 lung areas.

Possible limitations of the study could be in the selection of part of the study population, cause some patients were included with compatible COVID-19 symptoms diagnosis without a PCR confirmatory test. The process of extraction of some variables could also be a limitation, independent of how they have been coded as such by the Medical Health Record (MHR) story. This may have led to a possible reduction in the majority of patients with COVID-19 not coded but who had compatible symptoms. The lack of clinical characterization of each cohort (TC and VC) regarding comorbidities could be a limitation cause there may be interrelationships between different variables among them, which could interfere when establishing associations. To achieve this, each variable will be analyzed independently of the ultrasound findings and each other. Finally, the variability between observation during the ultrasound or testing technique, could be a limitation. However, this limitation is reduced by ensuring the general practitioners abilities demonstrated by general measurements in the performance of lung ultrasound.

To solve the logistical challenge that this study represents, we established: -Maximum coordination with the health management of the participating centres. -Maximum synergy with the health care circuit already established for the diagnosis and follow-up of COVID-19 patients in PHC. -The scanning technique and system will be common among all participating professionals, following the recommendations of the International Consensus of Ultrasound experts. -Pulmonary ultrasound information will be collected through the Data Collection Form. -Patients will be classified according to ultrasound stratification provided by the working group of the Catalan Society of Family and Community Medicine based on the severity of the lung involvement depending on the type of injury, location and extent affected.

## Conclusions

The results of this study will contribute to the determination of the pathologic ultrasound patterns in COVID-19 pneumonia, based on the number of lung areas affected with the presence of more than 3 B lines, pulmonary condensations or the irregularity of pleural effusion patterns. In addition, performing a lung ultrasound in patients with COVID-19 in primary care, based on their potential to identify the severity of lung impairment and its location, might contribute to identify patients at risk of being hospitalized or being admitted at the ICU, allowing a more personalized management. It will allow selecting patients with a risk profile to implement a stricter follow-up with the use of lung ultrasound as a support tool in the clinic. So far, in this study we describe the clinical prognosis in relation to certain ultrasound patterns.

## Supplementary Information

Supplementary Material 1.

## Data Availability

The datasets generated and/or analysed during the current study are not publicly available due to institutional policies, but are available from the corresponding author on reasonable request.

## Abbreviations

ARDS: Acute Respiratory Distress Syndrome
ARIs: Acute Respiratory Infections
AUC: Area Under the Curve
CAMFIC: Catalan Society of Family and Community Medicine
CEIC: Clinical Research Ethics Committee
CI: Confidence Interval
CT: Computed Tomography
DCR: Data Collection Report
HER: Electronic History Record
HR: Hazard Ratio
ICD-10: International Classification of Diseases
ICU: Intensive Care Unit
IDIAPJGol: Institut Universitari per a la recerca a l'Atenció Primària de Salut Jordi Gol i Gurina
IRB Lleida: Biomedical Research Institute of Lleida
LUS: Lung Ultrasounds
MHR: Medical Health Record
NPV: Negative Predictive Value
PC: Primary Care
PPV: Positive Predictive Value
ROC: Receiver Operating Characteristic
RT-PCR: Reverse Transcription Polymerase Chain Reaction
SIDIAP: System for Research in Primary Care
TC: Training Cohort
VC: Validation Cohort
