# Supplementary material for: Clinical and ultrasound characteristics in patients with sars-cov-2 pneumonia, associated with hospitalization prognosis. e-covid project
Source: BMC Pulm Med. 2024 Dec 31;24:638. doi: 10.1186/s12890-024-03439-2 (PMC11686988; doi:10.1186/s12890-024-03439-2)
Supplement: Supplementary file 1 — Supplementary Material 1. [file 12890_2024_3439_MOESM1_ESM.pdf]

SUPPLEMENTARY FIGURES

Basal visit	Date: ID Patient:		
Age: Sex: ☐ Male ☐ Women Days onset symptoms: ☐ 1-3 days ☐ ≥ 4 days		Symptoms	☐ No ☐ Pleural pain ☐ Dyspnoea ☐ Vomits ☐ Diarrhea
Risk Factors: ☐ COPD ☐ Asthma ☐ CVD ☐ Immune disease ☐ Obesity ☐ DM	Heart rate: bpm	Breath rate: rpm	SatO2: %FiO2
Data chest Rx: ☐ Normal ☐ Interstitial focal ☐ Interstitial diffuse ☐ Condensation ☐ Pleural effusion			

RIGHT HEMITHORAX

1R	
+/++/+++	B lines
yes / no	pleural irregularity
yes / no	subpleural condensation
yes / no	condensation

2R	
+/++/+++	B lines
yes / no	pleural irregularity
yes / no	subpleural condensation
yes / no	condensation

3R	
+/++/+++	B lines
yes / no	pleural irregularity
yes / no	subpleural condensation
yes / no	condensation

4R	
+/++/+++	B lines
yes / no	pleural irregularity
yes / no	subpleural condensation
yes / no	condensation

5R	
+/++/+++	B lines
yes / no	pleural irregularity
yes / no	subpleural condensation
yes / no	condensation

6R	
+/++/+++	B lines
yes / no	pleural irregularity
yes / no	subpleural condensation
yes / no	condensation

9R Sedestation	
+/++/+++	B lines
yes / no	pleural irregularity
yes / no	subpleural condensation
yes / no	condensation

10R Sedestation	
+/++/+++	B lines
yes / no	pleural irregularity
yes / no	subpleural condensation
yes / no	condensation

☐ Pleural effusion ☐ Ultrasounds without alterations

LEFT HEMITHORAX

1L	
+/++/+++	B lines
yes / no	pleural irregularity
yes / no	subpleural condensation
yes / no	condensation

2L	
+/++/+++	B lines
yes / no	pleural irregularity
yes / no	subpleural condensation
yes / no	condensation

3L	
+/++/+++	B lines
yes / no	pleural irregularity
yes / no	subpleural condensation
yes / no	condensation

4L	
+/++/+++	B lines
yes / no	pleural irregularity
yes / no	subpleural condensation
yes / no	condensation

5L	
+/++/+++	B lines
yes / no	pleural irregularity
yes / no	subpleural condensation
yes / no	condensation

6L	
+/++/+++	B lines
yes / no	pleural irregularity
yes / no	subpleural condensation
yes / no	condensation

11L Sedestation	
+/++/+++	B lines
yes / no	pleural irregularity
yes / no	subpleural condensation
yes / no	condensation

12L Sedestation	
+/++/+++	B lines
yes / no	pleural irregularity
yes / no	subpleural condensation
yes / no	condensation

☐ Pleural effusion ☐ Ultrasounds without alterations

Stratification visit: ☐ 1.Stable patient, normal ultrasound ☐ 2.Stable patient, mild ultrasound abnormality ☐ 3.Stable patient, diffuse ultrasound abnormality (there may be associated condensation)
☐ 4.With O2 requirement and diffuse ultrasound disturbance ☐ 5.With O2 requirement, patched pattern with condensation
Place of ultrasound: ☐ Primary Health Center ☐ Home ☐ Others:
Destination: ☐ Home ☐ Hospital ☐ Others:

Figure 1. Basal variables.

48 h visit	Date:	ID Patient:	
Heart rate: bpm	Breath rate: rpm	SatO2:	%FiO2

RIGHT HEMITHORAX **LEFT HEMITHORAX**

1R		2R		1L		2L	
+/++/+++	B lines	+/++/+++	B lines	+/++/+++	B lines	+/++/+++	B lines
yes / no	pleural irregularity	yes / no	pleural irregularity	yes / no	pleural irregularity	yes / no	pleural irregularity
yes / no	subpleural condensation	yes / no	subpleural condensation	yes / no	subpleural condensation	yes / no	subpleural condensation
yes / no	condensation	yes / no	condensation	yes / no	condensation	yes / no	condensation

3R		4R		3L		4L	
+/++/+++	B lines	+/++/+++	B lines	+/++/+++	B lines	+/++/+++	B lines
yes / no	pleural irregularity	yes / no	pleural irregularity	yes / no	pleural irregularity	yes / no	pleural irregularity
yes / no	subpleural condensation	yes / no	subpleural condensation	yes / no	subpleural condensation	yes / no	subpleural condensation
yes / no	condensation	yes / no	condensation	yes / no	condensation	yes / no	condensation

5R		6R		5L		6L	
+/++/+++	B lines	+/++/+++	B lines	+/++/+++	B lines	+/++/+++	B lines
yes / no	pleural irregularity	yes / no	pleural irregularity	yes / no	pleural irregularity	yes / no	pleural irregularity
yes / no	subpleural condensation	yes / no	subpleural condensation	yes / no	subpleural condensation	yes / no	subpleural condensation
yes / no	condensation	yes / no	condensation	yes / no	condensation	yes / no	condensation

9R Sedestation		10R Sedestation		11L Sedestation		12L Sedestation	
+/++/+++	B lines	+/++/+++	B lines	+/++/+++	B lines	+/++/+++	B lines
yes / no	pleural irregularity	yes / no	pleural irregularity	yes / no	pleural irregularity	yes / no	pleural irregularity
yes / no	subpleural condensation	yes / no	subpleural condensation	yes / no	subpleural condensation	yes / no	subpleural condensation
yes / no	condensation	yes / no	condensation	yes / no	condensation	yes / no	condensation

☐ Pleural effusion ☐ Ultrasounds without alterations ☐ Pleural effusion ☐ Ultrasounds without alterations

Stratification visit: ☐1.Stable patient, normal ultrasound ☐2.Stable patient, mild ultrasound abnormality ☐3.Stable patient, diffuse ultrasound abnormality (there may be associated condensation)

☐4.With O2 requirement and diffuse ultrasound disturbance ☐5.With O2 requirement, patched pattern with condensation

Destination: ☐ Home ☐ Hospital ☐Others:

Figure 2. Variables at 48h from basal visit.

2 weeks visit	Date: ID Patient:		
Heart rate: bpm	Breath rate: rpm	SatO2:	%FiO2

RIGHT HEMITHORAX **LEFT HEMITHORAX**

1R	
+/++/+++	B lines
yes / no	pleural irregularity
yes / no	subpleural condensation
yes / no	condensation

2R	
+/++/+++	B lines
yes / no	pleural irregularity
yes / no	subpleural condensation
yes / no	condensation

1L	
+/++/+++	B lines
yes / no	pleural irregularity
yes / no	subpleural condensation
yes / no	condensation

2L	
+/++/+++	B lines
yes / no	pleural irregularity
yes / no	subpleural condensation
yes / no	condensation

3R	
+/++/+++	B lines
yes / no	pleural irregularity
yes / no	subpleural condensation
yes / no	condensation

4R	
+/++/+++	B lines
yes / no	pleural irregularity
yes / no	subpleural condensation
yes / no	condensation

3L	
+/++/+++	B lines
yes / no	pleural irregularity
yes / no	subpleural condensation
yes / no	condensation

4L	
+/++/+++	B lines
yes / no	pleural irregularity
yes / no	subpleural condensation
yes / no	condensation

5R	
+/++/+++	B lines
yes / no	pleural irregularity
yes / no	subpleural condensation
yes / no	condensation

6R	
+/++/+++	B lines
yes / no	pleural irregularity
yes / no	subpleural condensation
yes / no	condensation

5L	
+/++/+++	B lines
yes / no	pleural irregularity
yes / no	subpleural condensation
yes / no	condensation

6L	
+/++/+++	B lines
yes / no	pleural irregularity
yes / no	subpleural condensation
yes / no	condensation

9R Sedestation	
+/++/+++	B lines
yes / no	pleural irregularity
yes / no	subpleural condensation
yes / no	condensation

10R Sedestation	
+/++/+++	B lines
yes / no	pleural irregularity
yes / no	subpleural condensation
yes / no	condensation

11L Sedestation	
+/++/+++	B lines
yes / no	pleural irregularity
yes / no	subpleural condensation
yes / no	condensation

12L Sedestation	
+/++/+++	B lines
yes / no	pleural irregularity
yes / no	subpleural condensation
yes / no	condensation

☐ Pleural effusion ☐ Ultrasounds without alterations ☐ Pleural effusion ☐ Ultrasounds without alterations

Stratification visit: ☐1.Stable patient, normal ultrasound ☐2.Stable patient, mild ultrasound abnormality ☐3.Stable patient, diffuse ultrasound abnormality (there may be associated condensation)

☐4.With O2 requirement and diffuse ultrasound disturbance ☐5.With O2 requirement, patched pattern with condensation

Destination: ☐Home ☐Hospital ☐Others:

Figure 3. Variables at 2 weeks from basal visit.

4 weeks visit	Date: ID Patient:		
Heart rate: bpm	Breath rate: rpm	SatO2:	%FiO2

RIGHT HEMITHORAX **LEFT HEMITHORAX**

1R	
+/++/+++	B lines
yes / no	pleural irregularity
yes / no	subpleural condensation
yes / no	condensation

2R	
+/++/+++	B lines
yes / no	pleural irregularity
yes / no	subpleural condensation
yes / no	condensation

1L	
+/++/+++	B lines
yes / no	pleural irregularity
yes / no	subpleural condensation
yes / no	condensation

2L	
+/++/+++	B lines
yes / no	pleural irregularity
yes / no	subpleural condensation
yes / no	condensation

3R	
+/++/+++	B lines
yes / no	pleural irregularity
yes / no	subpleural condensation
yes / no	condensation

4R	
+/++/+++	B lines
yes / no	pleural irregularity
yes / no	subpleural condensation
yes / no	condensation

3L	
+/++/+++	B lines
yes / no	pleural irregularity
yes / no	subpleural condensation
yes / no	condensation

4L	
+/++/+++	B lines
yes / no	pleural irregularity
yes / no	subpleural condensation
yes / no	condensation

5R	
+/++/+++	B lines
yes / no	pleural irregularity
yes / no	subpleural condensation
yes / no	condensation

6R	
+/++/+++	B lines
yes / no	pleural irregularity
yes / no	subpleural condensation
yes / no	condensation

5L	
+/++/+++	B lines
yes / no	pleural irregularity
yes / no	subpleural condensation
yes / no	condensation

6L	
+/++/+++	B lines
yes / no	pleural irregularity
yes / no	subpleural condensation
yes / no	condensation

9R Sedestation	
+/++/+++	B lines
yes / no	pleural irregularity
yes / no	subpleural condensation
yes / no	condensation

10R Sedestation	
+/++/+++	B lines
yes / no	pleural irregularity
yes / no	subpleural condensation
yes / no	condensation

11L Sedestation	
+/++/+++	B lines
yes / no	pleural irregularity
yes / no	subpleural condensation
yes / no	condensation

12L Sedestation	
+/++/+++	B lines
yes / no	pleural irregularity
yes / no	subpleural condensation
yes / no	condensation

☐ Pleural effusion ☐ Ultrasounds without alterations ☐ Pleural effusion ☐ Ultrasounds without alterations

Stratification visit: ☐1.Stable patient, normal ultrasound ☐2.Stable patient, mild ultrasound abnormality ☐3.Stable patient, diffuse ultrasound abnormality (there may be associated condensation)

☐ 4.With O2 requirement and diffuse ultrasound disturbance ☐5.With O2 requirement, patched pattern with condensation

Destination: ☐ Home ☐ Hospital ☐ Others:

Figure 4. Variables at 4 weeks from basal visit.

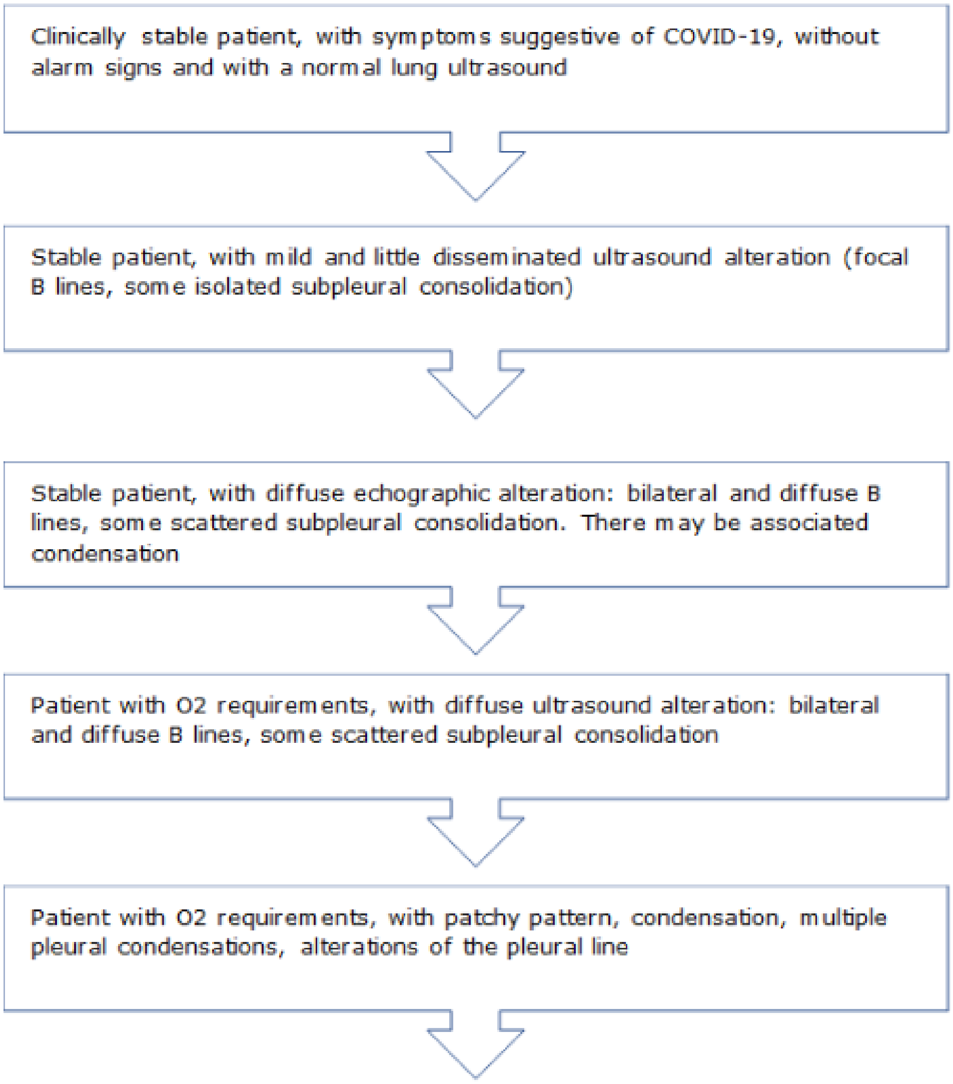

Figure 5. Severity findings and clinical-sonography risk stratification characterization classification provided by the Catalan Society of Family and Community Medicine (Camfic).

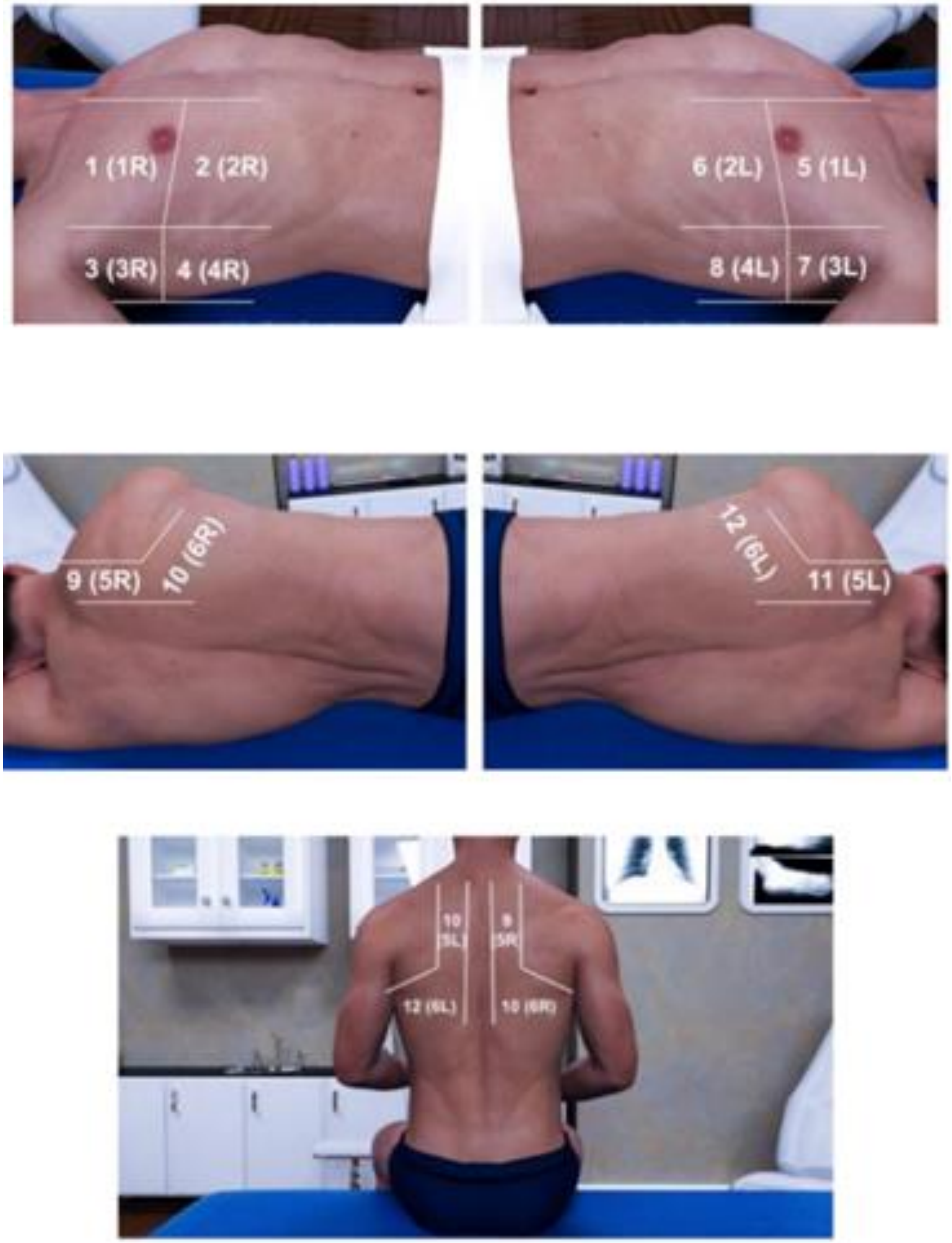

Figure 6. Thoracic areas studied.

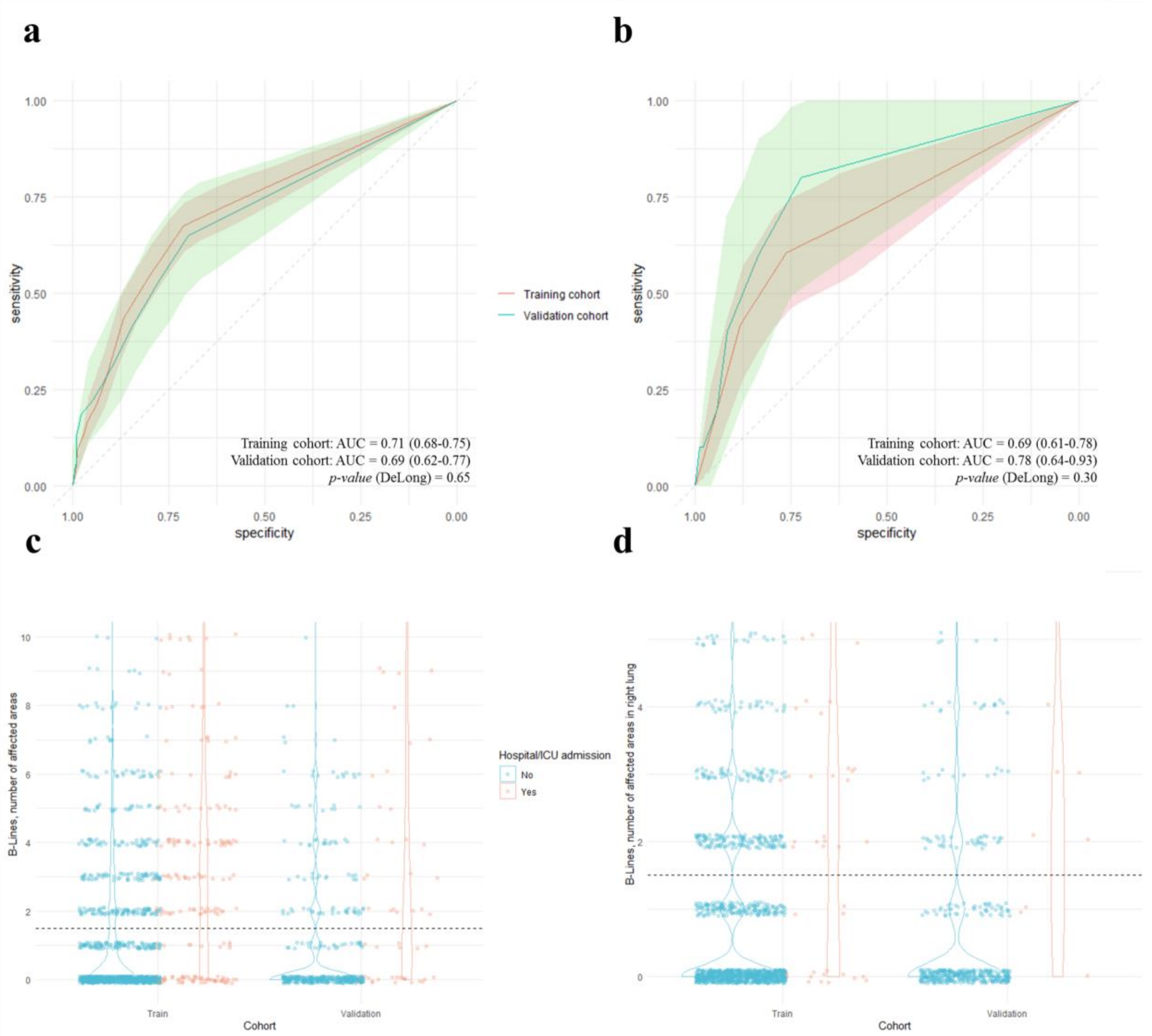

Figure 7. Predictive capacity for hospitalization and ICU admission based on lung ultrasound. a) ROC curve and c) violin plot of the hospitalization prediction model, using the total number of regions with B lines as a predictor. b) ROC curve and d) violin plot of the ICU admission prediction model, using the total number of regions with B lines in the right lung as a predictor. The dashed horizontal lines represent the optimal cut-off point for predicting hospitalization/ICU. admission.

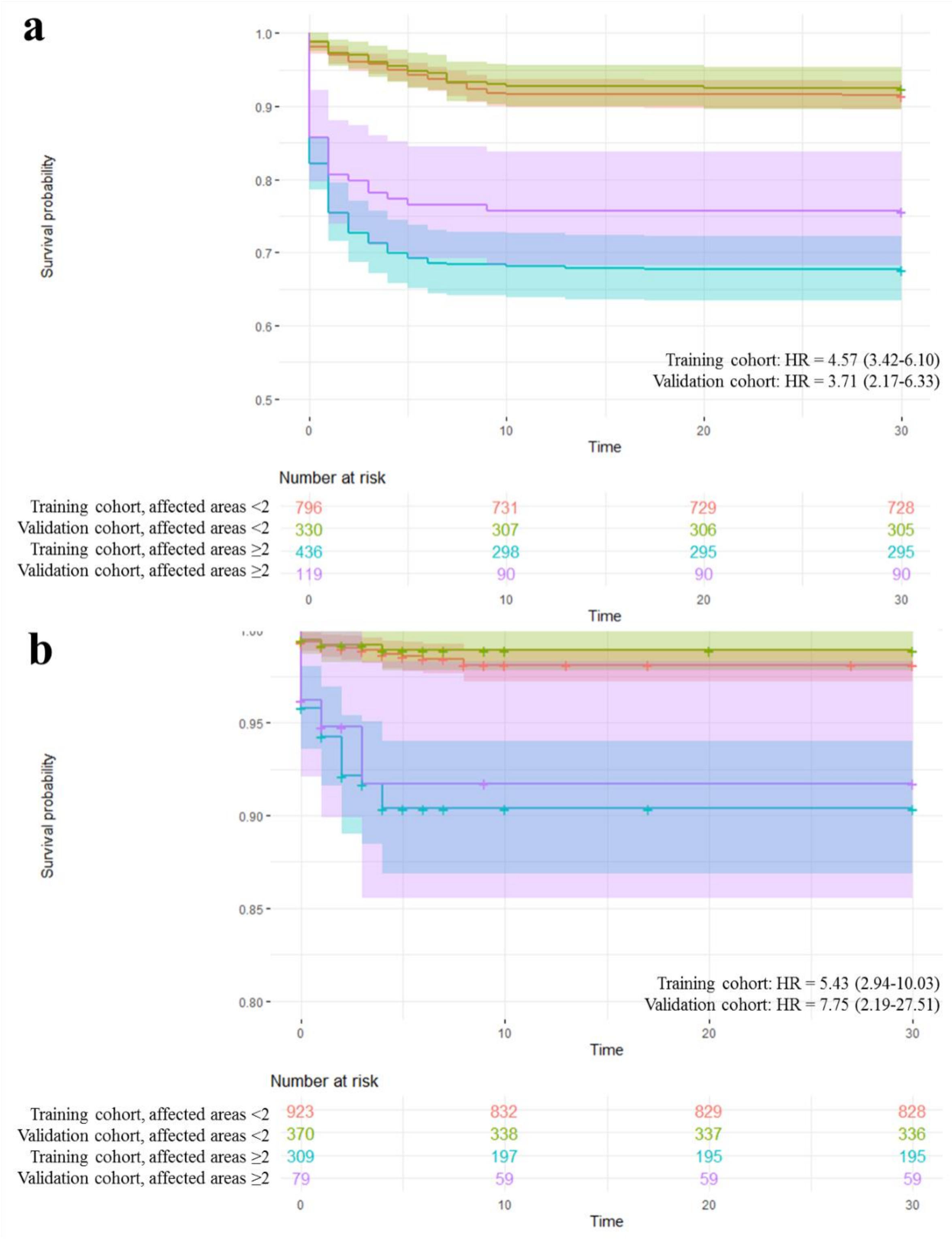

Figure 8: Survival models for hospitalization and ICU admission based on lung ultrasound. a) hospitalization prediction model, using the total number of regions with B lines ≥2 as a predictor. b) prediction model for ICU admissions, using the total number of regions with B lines in the right lung ≥2 as a predictor. HR: Hazard Ratio.

SUPPLEMENTARY TABLES

Table 1. Lung ultrasound findings.

+/++/+++	B lines
yes / no	pleural irregularity
yes / no	subpleural condensation
yes / no	condensation

+ indicates < 3 lines B (normal)
++ indicates > 3 B lines (mild interstitial)
+++ indicates white lung (diffuse interstitial)

Table 2. Sample description.

	Training (N=1232)	Validation (N=449)	*p-value*
Diagnostic ultrasound-days mean (SD)	2.40 (2.53)	2.34 (2.53)	0.708
Sex (female) n (%)	689 (55.9%)	241 (53.7%)	0.444
Age, mean (SD)	51.1 (17.6)	49.8 (18.7)	0.177
Vaccinated against COVID-19 (at least one dose)n (%)	27 (2.19%)	281 (62.6%)	<0.001
Hospitalization n (%)	209 (17.0%)	54 (12%)	**0.017**
Admission to ICU (intensive care unit) n (%)	43 (3.49%)	10 (2.23%)	0.249
Ultrasound-hospitalization days, mean (SD)	2.19 (3.49)	2.57 (3.69)	0.495
Ultrasound-ICU days, mean (SD)	3.66 (2.17)	6.86 (4.95)	0.140
Hospitalization days, mean (SD)	11.4 (12.3)	13.0 (17.2)	0.513
Days of admission to ICU, mean (SD)	3.14 (10.9)	4.50 (13.0)	0.48

Table 3. Performance of predictive models for hospitalization and ICU admission based on lung ultrasound.

	Hospital admission model performance							
	ROC AUC (95%CI)	HR (95%CI)	Cutoff	Accuracy (95%CI)	Sensitivity (95%CI)	Specificity (95%CI)	PPV (95%CI)	NPV (95%CI)
Training	0.71 (0.67-0.75)	1.24 (1.20-1.29)	≥2	70.54 (67.9-73.07)	67.46 (61.11-73.82)	71.16 (68.39-73.94)	32.34 (27.95-36.73)	91.46 (89.52-93.4)
Validation	0.69 (0.62-0.77)	1.25 (1.17-1.35)	≥2	74.39 (70.09-78.36)	53.7 (40.4-67)	77.22 (73.08-81.35)	24.37 (16.66-32.08)	92.42 (89.57-95.28)
	ICU admission model performance							
	ROC AUC (95%CI)	HR (95%CI)	Cutoff	Accuracy (95%CI)	Sensitivity (95%CI)	Specificity (95%CI)	PPV (95%CI)	NPV (95%CI)
Training	0.69 (0.61-0.78)	1.52 (1.32-1.74)	≥2	75.65 (73.15-78.02)	60.47 (45.85-75.08)	76.2 (73.78-78.62)	8.41 (5.32-11.51)	98.16 (97.29-99.03)

Validation	0.78 (0.64-0.93)	1.70 (1.28-2.25)	≥2	82.85 (79.04-86.22)	60 (29.64-90.36)	83.37 (79.89-86.85)	7.59 (1.75-13.44)	98.92 (97.87-99.97)
